# The Effect of Monoterpenes on Swarming Differentiation and Haemolysin Activity in *Proteus mirabilis*

**DOI:** 10.3390/molecules13123107

**Published:** 2008-12-15

**Authors:** Sergio Echeverrigaray, Lessandra Michelim, Ana Paula Longaray Delamare, Cristiane Paim Andrade, Sérgio Olavo Pinto da Costa, Jucimar Zacaria

**Affiliations:** 1 Research Laboratory of Microbiology, Institute of Biotechnology, University of Caxias do Sul, Rua Francisco Getúlio Vargas, 1130, Caxias do Sul, Brazil 95070-560; 2 Division of Infectious Diseases. General Hospital of Caxias do Sul, University of Caxias do Sul, Av. Prof Antonio Vignolli, 255, Caxias do Sul, Brazil95070-560; Email: lessandra@gmail.com (L. M.)

**Keywords:** Terpenoids, Cell differentiation, *Proteus mirabilis*, MIC

## Abstract

Urinary tract infection by *Proteus mirabilis* depends on several virulence properties that are coordinately regulated with swarming differentiation. Here we report the antibacterial and anti-swarming effect of seventeen terpenoids, and the effect of sub-inhibitory concentrations of five selected terpenoids on swarming, biofilm formation and haemolysin activity. The results showed that all the terpenes evaluated, particularly oxygenated terpenoids, inhibited *P. mirabilis* with MIC values ranging between 3 and 10 mg/L. Moreover, citral, citronellol and geraniol effectively inhibit *P. mirabilis* swarming in a dose dependent manner, reducing swimming/swarming cell differentiation and haemolysin activity at 1/10 MIC concentration. The inhibition of *P. mirabilis* swarming and virulence factor expression by selected oxygenated terpenoids suggest that essential oils with high concentration of these compounds have the potential to be developed as products for preventing *P. mirabilis* infections.

## Introduction

*Proteus mirabilis* is one of the most important pathogens associated with complicated urinary tract infections and bacteremia, affecting patients with anatomical abnormalities, immunodeficiency and long-term urinary catheterization [1, 2]. Urinary tract infection with *P. mirabilis* start with bladder colonization, causing bacteriuria and cystitis, and can ascend to the kidneys, leading to acute pyelonephritis, chronic inflammation, kidney stones and renal failure.

A prominent feature of *P. mirabilis* is the ability to swarm on agar plates and form highly ordered and terraced colonies with characteristic concentric rings. Swarming is process in which short vegetative swimming cells differentiate to long highly flagellated forms referred to as swarmer cells [1,3]. Swarmer cell differentiation depends on surface contact, inhibition of flagellar rotation, cell density and cell-cell signaling [4, 5].

Several potential virulence factors including haemolysin, swarming, adhesins, proteases, and ureases, may be responsible for the pathogenicity of *P. mirabilis* [6,7,8]*.* The expression of virulence factors, including haemolysin, urease and protease, and the ability to invade human urothelial cells, is coordinately upregulated during swarming [5, 8,9,10]. Numerous compounds have been reported to prevent *P. mirabilis* swarming “*in vitro*”. Among these are charcoal and barbitone [11], urea [12], ethanol and sodium azide [13], *p*-nitrophenylglycerol [12, 14, 15], fatty acids [16], and resveratrol [17].

Terpenes are secondary metabolites, represented by hemiterpenes, monoterpenes, sesquiterpenes and their terpenoid derivatives. These isoprenoid compounds are the main constituents of essential oils, being responsible for their aroma or flavor. Essential oils are obtained from spices, aromatic herbs, fruits, and flowers. Generally, the oil composition is a balance of various compounds, although in many species one constituent may prevail over all others [18].

Terpenes and terpenoids are active against bacteria, fungi, viruses, and protozoa [18]. The antibacterial action of terpenes is not fully understood but is speculated to involve membrane modifications resulting in alterations of membrane permeability and in leakage of intracellular materials [19]. Classified as Generally Recognized As Safe (GRAS), essential oils are used in food, cosmetics and pharmaceuticals, and have gained special interest because of the resistance to antibiotics that microorganisms have acquired [20,21].

In this study we evaluated the antimicrobial activity of several monoterpenes, and the effect of sub-inhibitory concentrations of these compounds on swarming, and haemolysin activity of *Proteus mirabilis*.

## Results and Discussion

The anti-bacterial activity of seventeen monoterpenes against *P. mirabilis* L68, as revealed by their MIC values, is summarized in Table 1. The results showed that monoterpenes exhibited anti-bacterial activity with varying magnitudes. Citral, citronellol, geraniol, α-terpineol, terpinene-4-ol, linalool, and pulegone were the most effective monoterpenes against *P. mirabilis* L68, with MIC values between 3 and 5 mg/L. This results were confirmed with five other isolates of *P. mirabilis* and two isolates of *P. vulgaris* (data not show), indicating that these MIC values are representative of the antimicrobial activity of these monoterpenes against *Proteus*.

**Table 1:** Minimum inhibitory concentration (MIC) and the effect of 1/10 MIC concentration of monoterpenes on the swimming/swarming behavior of *P. mirabilis* L68.

Treatments	MIC (mg/L)	Colony diameter (mm)*^1^	Nº of concentic rings	Second ring width (mm)^1^
pulegone	5	53.2 ± 1.5^e^	5-6	3.8 ± 1.4^bc^
citronellol	3	23.7 ± 1.5^f^	5-6	1.0 ± 0.5^d^
citronellal	6	68.0 ± 0.6^b^	6	4.3 ± 0.3^b^
citronellyl acetate	>10	79.2 ± 3.5^b^	6	4.5 ± 0.9^b^
geraniol	3	22.5 ± 0.6^f^	3-4	0.8 ± 0.3^d^
bornyl acetate	10	71.3 ± 1.5^b^	6	4.3 ± 0.3^b^
α-terpinene	7.5	79.3 ± 3.5^ab^	5-6	6.1 ± 2.2^a^
α -terpineol	5	49.6 ± 1.0^d^	4-5	4.2 ± 0.4^b^
terpinene-4-ol	5	62.5 ± 1.0^c^	5-6	4.5 ± 1.3^b^
linalool	6	72.8 ± 1.7^b^	5-6	6.2 ± 1.2^a^
linallyl acetate	>10	82.1 ± 3.0^a^	6	4.5 ± 0.1^b^
limonene	10	82.0 ± 2.7^a^	6	5.4 ± 1.3^ab^
citral	5	41.0 ± 1.5^d^	4-5	2.8 ± 1.0^c^
mentone	>10	66.7 ± 1.7^c^	5-6	4.7 ± 1.7^b^
β-pinene	>10	79.5 ± 4.7^ab^	6	7.0 ± 3.6^a^
1,8-cineol	7.5	74.6 ± 2.5^b^	6	3.0 ± 0.9^c^
carveol	10	83.2 ± 1.7^a^	5-6	7.0 ± 2.3^a^
Control	-	84.2 ± 3.5^a^	6	6.5 ± 2.2^a^

^*^ Colony diameter in LB swarming plates with 1/10 MIC concentration (0.3 to 1 mg/L). Data were obtained after 24h at 37ºC; ^1^Means followed by the same letter are not significantly different according to Tukey´s test (p=0.05).

MIC values obtained in the present work are in accordance with previous reports in which the antibacterial effect of essential oils extracted from several plants were determined [22]. Moreover, the seven most effective compounds against *P. mirabilis*, as determined by their MIC values, are oxygenated monoterpenes considered as broad spectrum antibacterial substances [23].

The effect of terpenes on *Proteus* (*P. mirabilis*, *P. vulgaris* and *P. penneri*) swarming behavior was indirectly reported by Mansouri *et al.* [24]. These authors showed that subinhibitory concentrations of the essential oils of *Ferula gumosa*, *Lavandula officinalis*, and *Zataria multiflora* modified or inhibited swarming.

**Figure 1:**
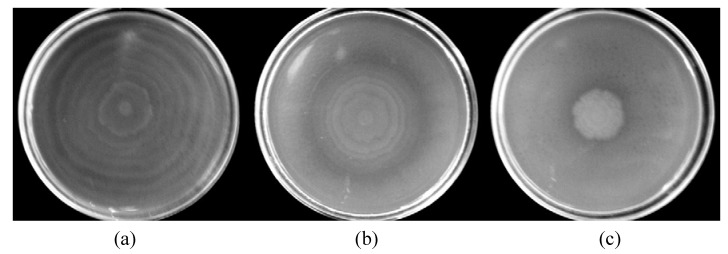
Swarming of *Proteus mirabilis* L68 on LB swarming agar plates without terpenes (a), and with 0.5 mg/L of α-terpineol (b) and 0.3 mg/mL citronellol (c). Incubation period of 24 h at 37ºC.

To evaluate the effect of the monoterpenes on *P. mirabilis* swarming behavior, LB swarming agar plates containing 1/10 MIC concentration of each compound were seeded with a drop (5 μL) of a stationary culture of wild-type L68 strain. These experiments showed that eight out of 17 compounds tested inhibited swarming significantly (Table 1). Swarming inhibition was evidenced by a reduction on colony diameter, the number of concentric rings, and ring width (Table 1 and Figure 1). The reduction of the number of concentric rings and ring width are first indicatives of the interference of sub-inhibitory concentrations of terpenoids on swimming/swarming cell differentiation and swarming cell motility. The eight compounds that effectively inhibit swarming were oxygenated monoterpenes. Four of them (citronellol, geraniol, α-terpineol, and terpinene-4-ol) are oxygenated compounds with hydroxyl groups, one is a cyclized monoterpenes alcohol (1,8-cineol), and three are monoterpene esters (citronellal, citral and pulegone).

Comparison between the effect of citronellol, citronellal and citronellyl acetate, linalool and linallyl acetate, and geraniol and citral, indicate that hydroxyl group is important in the antibacterial activity and swarming inhibition of *P. mirabilis* by monoterpenes (Table 1). In general, the antimicrobial activity of a compound increases with the presence of an oxygen containing functional group, indicating a relationship between structure and biological activity [25, 26].

**Figure 2:**
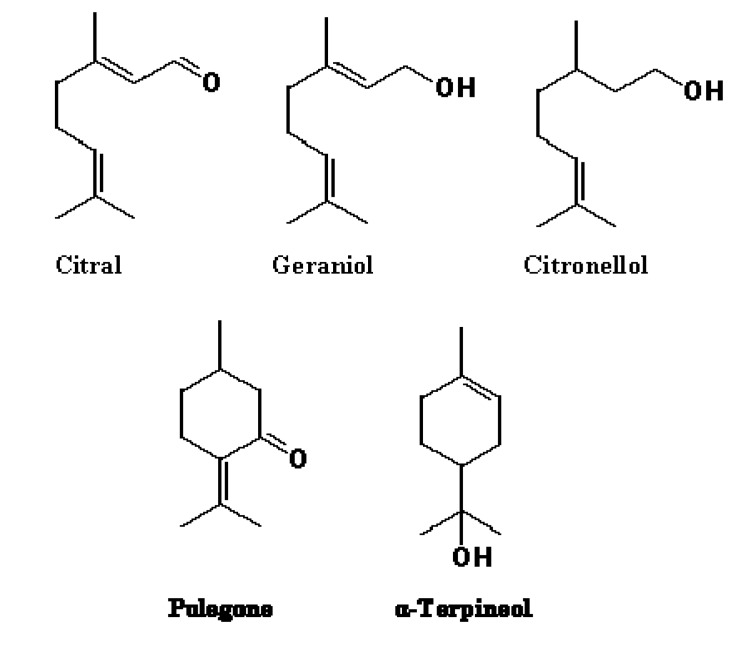
Chemical structures of monoterpenes with pronounced effect on swarming differentiation.

**Figure 3:**
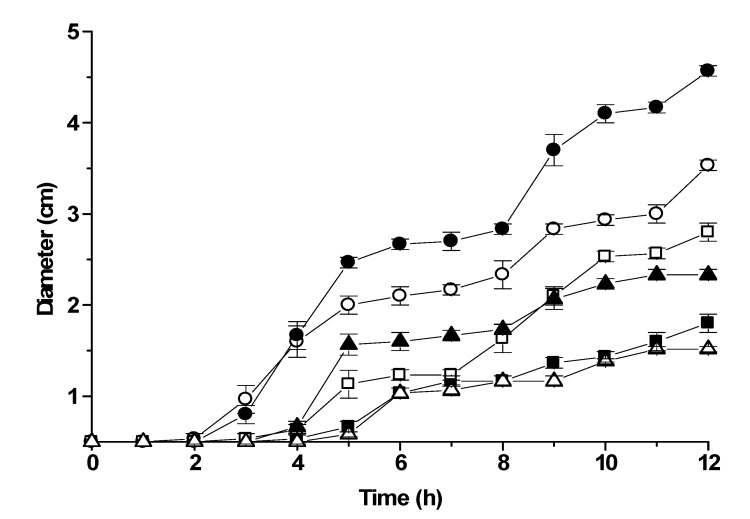
Effect of selected monoterpenes on the swarming behavior of *Proteus mirabilis* (L68 wild strain): ● control (no terpenes), ○ pulegone (0.5 mg/L), ▲ citral (0.5 mg/L), 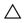 geraniol (0.3 mg/L), ■ citronellol (0.3 mg/L), □ α-terpineol (0.5mg/L). The data represent the averages of colony diameter of three independent experiments with standard deviations.

For further studies, we selected five terpenoids (citral, citronellol, α-terpineol, geraniol, and pulegone, Figure 2) that exhibited significant reduction of *P. mirabilis* swarming. As can be observed in Figure 3, these terpenoids (0.3 to 0.5 mg/L) reduce colony expansion over time. Moreover, swimming/swarming cyclic differentiation was not observed in the presence of citronellol and geraniol. To test whether swarming inhibition was dose dependent, the selected monoterpenes at 0.06, 0.12, 0.25, 0.5, and 0.75 mg/L were added to LB swarming agar plates, and colony diameter evaluated after 18 h at 37ºC.

**Figure 4:**
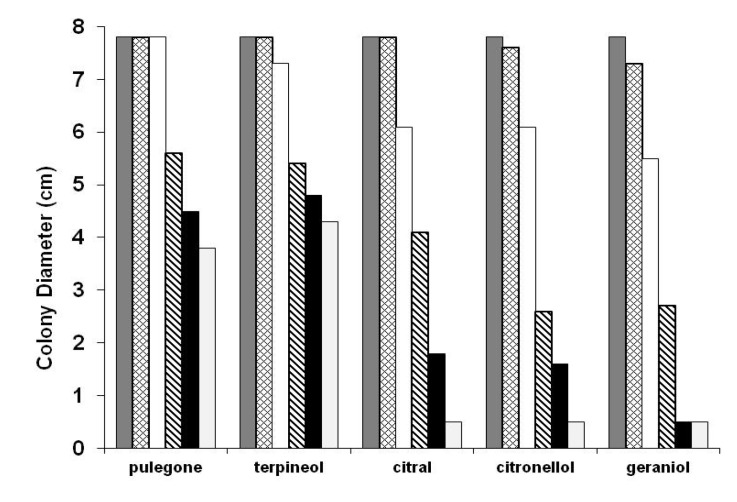
Histogram showing the swarming migration of *P. mirabilis* in the presence of different concentrations of glycerol and selected monoterpenes. Data represent the average of three independent experiments: 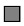 Control;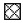 0.06 mg/L;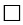 0.12 mg/L;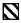 0.25 mg/L;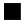 0.50 mg/L and 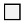 0.75 mg/L.

As shown in Figure 4, swarming of *P. mirabilis* was inhibited in a dose dependent manner by the five terpenoids. Complete inhibition was observed in plates containing 0.25 and 0.75 mg/L of geraniol, and 0.75 mg/L of citral and citronellol. Similar dose dependent inhibition of swarming behavior on wild-type strains of *P. mirabilis* was previously verified for *p*-nitrophenylglycerol [14], fatty acids [16], and resveratrol [17].

To test if the inhibitory effect of terpenoids on swarming arise from a toxic effect on the bacteria, we evaluated whether 1/10 MIC concentration of selected terpenoids affected the growth rate of *P. mirabilis* L68. *P. mirabilis* L68 growth was not significantly reduced by citral, citronellol and geraniol, and regardless the presence of the terpenoids, the bacteria reach stationary phase with similar cell density after 16 h (approx. 10^9 ^cells/mL), indicating that terpenoids effect on swarming is unlikely to be due to cell growth inhibition.

**Figure 5:**
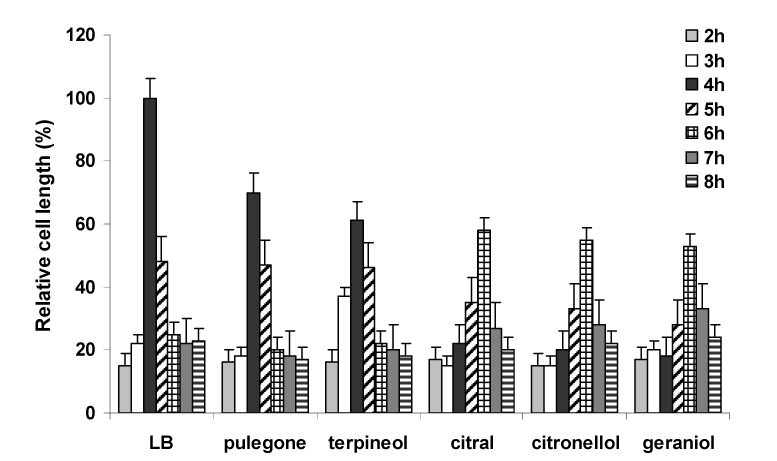
Effect of selected monoterpenes on the cell length of *P. mirabilis* L68. The concentration of monoterpenes added were 0.3 mg/L for citronellol and geraniol, and 0.5 mg/L for citral, pulegone and α-terpineol. Data represent the average of three independent experiments.

The swimming/swarming behavior of *P. mirabilis* involves the coordinate differentiation of short, motile, vegetative cells with few peritrichous flagella into long, multi-flagellated, swarm cells [5]. Cell differentiation was evaluated in the presence or absence of terpenoids on LB swarming agar. As can be observed in Figure 5, in the absence of terpenoids, *P. mirabilis* differentiated into long cell after 4 h, undergoing a rapid reduction on cell length after this period.

Similar behavior was observed in the presence of α-terpineol and pulegone, but elongated cell were detected only 2 h later in media supplemented with citral, citronellol or geraniol. Moreover, although the five selected terpenoids reduced swarming cell length, cells were particularly shorter in the presence of citral, citronellol and geraniol. These data indicate that terpenoids inhibited swarming by interference in swimming/swarming cell differentiation.

**Figure 6:**
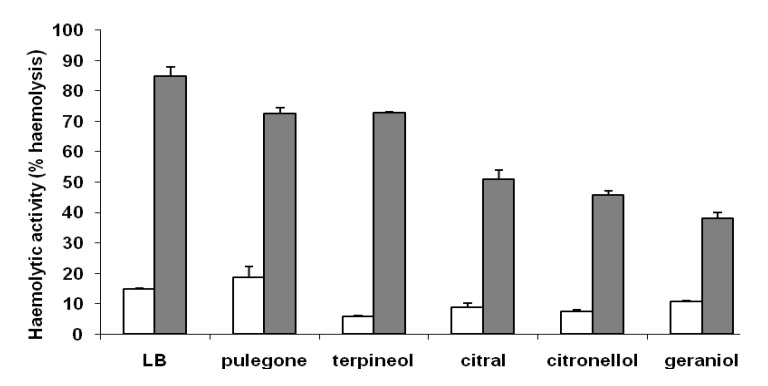
Effect of selected monoterpenes on the haemolysin activity of *P. mirabilis* L68. The concentration of monoterpenes added were 0.3 mg/L for citronellol and geraniol, and 0.5 mg/L for citral, pulegone and α-terpineol. Data represent the average of three independent experiments. Vegetative cells (white bars), Swarming cells (gray bars).

Expression of haemolysin, protease, and urease activities in *P. mirabilis*, are regulated coordinately with swarming differentiation [14, 15]. Typical swimming and swarming cells from the cell length experiments (Figure 4) were collected and evaluated for cell membrane-associated haemolysin activity, an important virulence factor in *P. mirabilis* [9]. As shown in Figure 6, *P. mirabilis* swarming cells expressed highest haemolysin activities. However, in the presence of monoterpenes, this activity was lower than that of the control. The reduction in haemolysin activity was directly correlated with the reduction in swarming cell length (R^2^=0.72), indicating that terpenoids affect cell differentiation, and consequently, haemolysin activity. This relation was observed for other compounds, as nitrophenylglycerol [14], fatty acids [16], and resveratrol [17].

Terpenes and their oxygenated products are relatively inexpensive, stable, abundant, and natural products with high concentration of these compounds, essential oils, are considered as Generally Recognized As Safe (GRAS) [18]. The inhibition of swarming and virulence factor expression suggests that essential oils with high concentration these compounds have the potential to be developed as products for preventing *P. mirabilis* infections.

## Experimental

### Bacterial strain and growth conditions

The bacterial strain used in this study was the wild-type *Proteus mirabilis* L68, isolated from a patient with chronic urinary tract infection [27]. Bacteria were routinely cultured at 37ºC in Luria-Bertani (LB) medium.

### Terpenes and terpenoids

The seventeen monoterpenes (Table 1) used were purchased from Acros Organics, New Jersey, USA. The purity of these commercial compounds is >90%.

### MIC assay

The Minimal Inhibitory Concentration assay of terpenes was performed by the broth microdilution [28]. Briefly, serial dilutions of monoterpenes, previously diluted in dimethylsulfoxide (DMSO), were prepared in sterile LB medium in 96-well microtiter plates. Freshly grown bacterial suspensions in LB were standardized to 10^8^ CFU/ml, and added to the wells (10 μL). The last row containing only the serial dilution of essential oils without bacteria was used as negative control. After incubation at 37ºC for 24 h the first well without turbidity was determined as the minimum inhibitory concentration (MIC). Controls without and with DMSO at the same concentration used to dilute the terpenes were included.

### Swarming behavior assay

The swarming migration distance assay was performed as described by Liaw *et al.* [15, 16]. Briefly, an overnight *P. mirabilis* L68 culture (5 μL) was inoculated centrally onto the surface of dried LB swarming agar (1.5%) plates without or with different monoterpenes. The plates were incubated at 37ºC, and the swarming migration distance was assayed by measuring the swarm fronts of the bacterial cells and recording progress at 60 min intervals. Controls plates without and with DMSO at the same concentration used to dilute the terpenes were included.

### Measurement of cell length

Measurement of cell length was performed as described by Liaw *et al.* [16], with modifications. Briefly, stationary-phase LB cultures (200 μL) were spread onto LB agar plates without and with appropriate terpenes (1/10 MIC concentration) and incubated at 37ºC. Cell from the entire surface of agar plates were harvested with saline (2 mL) at 1 h intervals. Bacterial cells were fixed and stained with 0.18% safranin solution (Merck, Germany), examined by light microscopy (Bioval, China) at a magnification of 1000x, and digitalized using a Samsung digital camera. The lengths of 100 cells in each sample were determined, and the average was calculated. Control plates without and with DMSO at the same concentration used to dilute the terpenes were included.

### Membrane-associated haemolysin

Swimming and swarming cells collected from agar plates were washed with saline and adjusted to a DO_600_= 0.7. Cell membrane-associated haemolysin activity was assayed as previously described by Koronakis *et al.* [29] and Liaw *et al.* [16]. Briefly, bacterial suspension (50 µL) was mixed with a 2% erythrocyte suspension (450 µL) in 0.85% NaCl plus 20 mM CaCl_2_ and incubated at 40ºC for 15 min. Haemolytic activity was measured by the release of haemoglobin (A_543_) using a 100% positive haemolysin-positive reference.
